# Current opinions concerning the restoration of endodontically treated teeth: basic principles

**Published:** 2009-04-25

**Authors:** M Greabu, M Battino, M Mohora, A Totan, A Didilescu, T Spinu, C Totan, D Miricescu, R Radulescu

**Affiliations:** * Department of Biochemistry, Faculty of Dental Medicine,‘Carol Davila‘ University of Medicine and Pharmacy, Bucharest, Romania Romania; ** Institute of Biochemistry, Faculty of Medicine, Universita Politecnica delle Marche, Ancona, Italy Romania; *** Department of Biochemistry, Faculty of General Medicine, ‘Carol Davila‘ University of Medicine and Pharmacy, Bucharest, Romania Romania; **** Department of Anatomy and Embryology, Faculty of Dental Medicine, ‘Carol Davila‘ University of Medicine and Pharmacy, Bucharest, Romania Romania; ***** Department of Fixed Prosthodontics and Dental Occlusion, Faculty of Dental Medicine, ‘Carol Davila‘ University of Medicine and Pharmacy, Bucharest, Romania Romania; ****** Department of Oral Surgery, Faculty of Dental Medicine, ‘Carol Davila‘ University of Medicine and Pharmacy, Bucharest, Romania Romania

**Keywords:** saliva, diagnosis, systemic diseases, oral diseases, sialochemistry

## Abstract

Saliva, the most available and non–invasive biofluid of the human body, permanently ‘bathes’ the oral cavity and is trying to cope with an ever–changing milieu. The oral cavity, a very complex and unique milieu due to its dual function, is the only place in the body where the mineralized tissue is exposed to the external environment in which there are complex interactions between various surfaces: host soft and hard tissues, food, air, and microorganisms. Saliva includes a large number of inorganic and organic compounds, which act as a ‘mirror of the body's health.’ In addition to its other functions, saliva could constitute the first line of defense against oxidative stress. Due to its composition and functions, saliva could have a significant role in controlling and/or modulating oxidative damages in the oral cavity. As a diagnostic fluid, saliva offers distinctive advantages over serum. Furthermore, saliva may provide a cost–effective approach for the screening of large populations. Gland–specific saliva can be used for diagnosis of pathology specific to one of the major salivary glands. Whole saliva, however, is most frequently used for diagnosis of systemic diseases.

As we enter the era of genomic medicine, sialochemistry will play an increasingly important role in the early detection, the monitoring and progression of the systemic and oral diseases. We reviewed the current data within literature and of our research concerning clinical potential of the saliva.

## Introduction

Saliva is derived from several types of salivary glands. Each type of salivary gland secretes saliva with characteristic composition and properties. The secretions from these different glands have been shown to differ considerably, to be complex in composition and to be affected by different forms of stimulation, time of day, diet, age, gender, a variety of disease states, and several pharmacological agents [1,4]. Whole saliva is a mixed fluid that is derived predominantly from 3 pairs of major salivary glands: the parotid, the submandibular, and the sublingual glands. Approximately 90% of total salivary volume results from the activity of these 3 pairs of glands, with the bulk of the remainder from minor salivary glands located at various oral mucosal sites. The whole saliva also contains gingival crevicular fluid (GCF), mucosal transudations, expectorated bronchial and nasal secretions, serum and blood derivatives from oral wounds, bacteria, and bacterial products, viruses and fungi, desquamated epithelial cells, other cellular components, and food debris.

Serum constituents that are not part of the normal salivary constituents (i.e., drugs and hormones) can reach saliva by several ways: intracellular (through passive transfer, by diffusion) and extracellular (ultrafiltration) [4, 6]. Serum constituents are also found in whole saliva as a result of GCF outflow. Depending on the degree of the inflammation in the gingiva, GCF is either a serum transudation or, more commonly, an inflammatory exudation that contains serum constituents.

Saliva can be collected with or without stimulation [7]. The best two ways to collect whole saliva are the draining method, in which saliva is allowed to drip off the lower lip, and the spitting method, in which the subject expectorates saliva into a test tube [8]. Let us analyze saliva.

## Salivary Composition

Saliva is a clear, slightly acidic (pH 6– 7) liquid.

### Inorganic components

The most abundant component in saliva is water(approximately 99%), followed by ions Na^+^,Cl^–^ Ca^2+^,K^+^,HCO_3_^–^ ,H_2_PO_4_^–^ , F^–^ , I^–^ ,Mg^2+^, thiocyanate. The ionic composition of saliva is different from the plasma although derived from it. [9]. The hypotonicity facilitates taste sensitivity and hydrates various organic compounds that form a protective coating on the oral mucosa. Resultant bicarbonate serves as a buffering agent and calcium and phosphate neutralize acids that would otherwise compromise tooth mineral integrity [9,10].

### Organic components

Saliva includes a large number of organic compounds such as: urea, ammonia, uric acid, glucose, cholesterol, fatty acids, mono–,di–, and triglycerides, phosphor and neutral lipids, glycolipids, amino acids, steroid hormones and proteins that aid in the protection of oral cavity tissues, including mucins, amylases, agglutinins, glycoproteins, lysozymes, peroxidases, lactoferrin and secretory IgA. Non–immune factors include lactoferrin, lysozyme, myeloperoxidase, histatins, cystatins, mucin G1 and G2, and defensins [11–17]. In addition, these macromolecules form a viscoelastic mucosal coat and tooth enamel pellicle and aggregate and cleanse bacteria and debris from the oral cavity [18]. Saliva contains a variety of antimicrobial constituents and growth factors [19,20].

There is a strong relationship functions–constituents of saliva and a number of salivary proteins participate in more than one function. Function of saliva are: digestion, mastication and deglutition, protective (antifungal, antibacterial, antiviral activity, lubricant and buffering agent), defense (spiting and oxidative stress) ,drug testing, water balance, excretion, speaking, denture retention, tasting, chemical communication (kissing  or infant salivating represents a lovable sight to a parent) [2,3,,21–23]. It is probably surprising for most people to learn that saliva has been used in diagnostics for more than two thousand years. Ancient doctors of traditional Chinese medicine have concluded that saliva and blood are ‘brothers’ in the body and they come from the same origin. It is believed that changes in saliva are indicative of the wellness of the patient.

Saliva offers some distinctive advantages [1,22, 24–26]: smaller sample aliquots, the possibility of a dynamic study, greater sensitivity, non–invasive, stress free and easy collection procedure, a good cooperation with patients, the possibility to collection somewhere and anywhere, no special equipment and not a trained technician are needed for collection, correlation with levels in blood, potentially valuable for children and older adults, more accurate than blood for detection of many oral and systemic diseases, may provide a cost–effective approach for the screening of large populations, could eliminate the potential risk of contracting infectious disease for both a technician and the patient.  

Advances in the use of saliva as a diagnostic fluid have been affected by current technological developments: enzyme–linked fluorescence technique, Western blot assays, polymerase chain reaction (PCR).

### Systemic Diseases

Salivary gland and the composition of saliva are affected directly or indirectly and these changes may contribute to the diagnosis and early detection of these diseases. 

The abnormal secretions present in **cystic fibrosis CF** caused clinicians to explore the usefulness of saliva for the diagnosis of the disease. Most studies agree that the saliva of CF patients contains increased calcium (resulted in a calcium–protein aggregation which caused turbidity of saliva and phosphate levels that could explain a higher occurrence of calculus, more neutral lipids, phospholipids and glycolipids, as a consequence for the altered physico–chemical properties of saliva in this disease, elevationsin urea, uric acid, and total protein, especially in submandibular saliva [27–33]. Saliva from CF patients was found to contain an unusual form of epidermal growth factor (EGF and abnormally elevated levels of prostaglandins E2 (PGE2) [33,34]. Early morning salivary levels of 17–hydroxyprogesterone (17–OHP) were reported to be an excellent screening test for the diagnosis of non–classic 21–hydroxylase deficiency [35].

**Sjogren's syndrome (SS)**, an autoimmune exocrinopathy of unknown etiology, a reduction in lachrymal and salivary secretions is observed, associated with keratoconjunctivitis sicca and xerostomia. Sialochemistry may also be used to assist in the diagnosis of SS: increased concentrations of sodium and chloride, elevated levels of IgA, IgG, lactoferrin, albumin, inflammatory mediators–i.e., eicosanoids, PGE2, thromboxane B2, and interleukin–6–have been reported, and a decreased concentration of phosphate. Reduced salivary flow is of clinical importance and can lead to a variety of oral signs and symptoms, such as progressive dental caries, fungal infections, oral pain, and dysphagia. Dentists are normally the first to encounter these patients [36–42].

Salivary analysis is more accurate than blood for detection of **cancer**.

It is well known that for most cancers, successful treatment depends on early detection and successful prevention depends on the accurate evaluation of risk. One of the most important missions of the medicine is to translate newly emerging molecular knowledge into practical clinical tests to detect cancer and cancer risk [24–26,43].

p53 is a tumor suppressor protein which is produced in cells exposed to various types of DNA–damaging stress. Inactivation of this suppressor through mutations and gene deletion is considered a frequent occurrence in the development of human cancer. The p53 antibodies can be detected in sera of patients with different types of malignancies in the saliva of patients diagnosed with oral squamous cell carcinoma (SCC) [44–48].

Elevated levels of salivary defensins, peptides that possess antimicrobial and cytotoxic properties founded in the azurophil granules of polymorphonuclear leukocytes (PMNs) were detected in patients with oral SCC [49–51].

Many studies indicate that there exist differences in patterns of mRNA expression in saliva that would indicate the presence of a developing SCC. Salivary mRNA may serve as a chemical signature that a particular gene has been expressed. Aberrant gene promoter methylation of DNA derived from exfoliated oral mucosal cells sampled from whole saliva has also been reported suggesting that this may form the basis of a screening test [24]. This has proven useful for both biomarker profiling and forensic identification.

Elevated levels of recognized tumor markers c–erbB–2 (erb) and cancer antigen 15–3 (CA15–3) were found in the saliva of women diagnosed with breast carcinoma, as compared with patients with benign lesions and healthy controls and thus appears to hold greater promise for the early screening and detection of breast cancer. CA 125 is a tumor marker for epithelial ovarian cancer. Elevated salivary levels of CA 125 were detected in patients with epithelial ovarian cancer [52,53].

**In cardiovascular diseases**, a major cause of death world–wide, if salivary amylase levels were low in postoperative patients with ruptured aortic aneurysm, there was an associated increase in mortality, Therefore, salivary amylase appears to be a more direct and simple end point of catecholamine activity than changes in heart rate when evaluating patients under a variety of stressful conditions [1,25,26].

Saliva can be analyzed as part of the evaluation of **endocrine function** [54]. Measurements of salivary hormone levels are of clinical importance if they accurately reflect the serum hormone levels or if a constant correlation exists between salivary and serum hormone levels [1,24,25]. Monitoring salivary testosterone levels may also be useful in behavioral studies of aggression, depression, abuse, and violent and antisocial behavior [55–57].

Saliva levels of steroid hormones reflect the free, and thus active, level of these hormones (while most blood measurements reflect the total level). Currently, the following steroids can be accurately assessed in saliva: aldosterone, insulin, cortisol, dehydroepiandrosterone, estradiol, estriol, progesterone and testosterone.

### Infectious diseases

#### Viral diseases

Saliva is superior to serum and urine to both sensitivity and specificity in testing for HIV infection, human herpes virus, cytomegalovirus, Epstein–Barr virus, hepatitis C virus [1,2,24,25]. The basis for many diagnostic tests in virology is the antibody response to infection. Saliva contains immunoglobulins (Ig) that originate from two sources: the salivary glands and serum. The predominant Ig in saliva is secretory IgA (sIgA), which is derived from plasma cells in the salivary glands, and constitutes the main specific immune defense mechanism in saliva [58–60]. In contrast, salivary IgM and IgG are primarily derived from serum via GCF, and are present in lower concentrations in saliva than is IgA. Antibodies against viruses and viral components can be detected in saliva and can aid in the diagnosis of acute viral infections, congenital infections, and reactivation of infection. Saliva was found to be a useful alternative to serum for the diagnosis of viral hepatitis, acute hepatitis A (HAV), B (HBV) [61–68].

For newborn infants, the salivary IgA response was found to be a better marker of rotavirus (RV) infection than the serum antibody response and can be used to monitor the immune response to vaccination and infection with RV [69].

Salivary IgA levels to HIV decline as infected patients become symptomatic. It was suggested that detection of IgA antibody to HIV in saliva might, therefore, be a prognostic indicator for the progression of HIV infection. In conclusion, collection and analysis of saliva offer a simple, safe, well–tolerated, and accurate method for the diagnosis of HIV infection. The non-invasive nature in which a sample is collected eliminates the risk of infection inherent in collecting blood samples [70–74].

#### Bacterial infections

Saliva is very useful for (diagnosis of Helicobacter pylori (associated with peptic ulcer), the detection of dental plaque–induced diseases, i.e. dental caries, gingivitis, periodontal disease [75–81]. In principle, this application of saliva–based diagnostics appears to be feasible3

**Detection, measurement, and monitoring of drugs**. Many analyses, including drugs of abuse, can be measured in saliva and oral fluids. Particularly useful where a ‘yes/no’ answer is required, oral fluid based tests find wide usage in detection of recreational drugs, including alcohol, amphetamines, barbiturates, benzodiazepines, cocaine, a variety of inhalants, lysergic acid diethylamide (LSD), marijuana, opioids, phencyclidine (PCP), and tobacco. The use of saliva for drug monitoring, and the detection of illicit drugs, has grown remarkably. currently, saliva can be used to detect and/or monitor nicotine, cannabinoids, cocaine, phencyclidine, opioids, barbiturates, diazepines, amphetamines and ethanol (most recently, law enforcement agencies have employed saliva–based tests for roadside evaluation of alcohol levels and in hospital emergency departments as a rapid means of determining whether impaired consciousness is related to alcohol intoxication) [24,26,82–84].

**Again and again about tobacco3** Tobacco usage or exposure (via ‘passive’ or ‘second–hand’ smoke) is now routinely measured by quantization oflevels of salivary nicotine that are similar clearance and half–life values as plasma. Monitoring levels of salivary nicotine has proven useful in monitoring self–reported compliance with smoking cessation programs. Salivary nicotine levels were found to be indicative of active and passive smoking [84–85]. Salivary thiocyanate was also found to be an indicator of cigarette smoking [86]; however, nicotine levels are considered the most reliable marker [87].An adequate intake may help smokers to avoid cigarette smoke induced oxidative damage and to prevent degenerative disease. The smoking causes the decrease in salivary important antioxidants levels and the loss of activity of salivary enzymes with antioxidant actions  can be considered as one of the mechanisms by which the toxic effects of CS initiate oral inflammatory diseases, promote precancerous transformations and destroy the oral cavity homeostasis [88–90].

**Oral Diseases** Evaluation of the quantity of whole saliva is simple and may provide information, which has systemic relevance [91]. Quantitative alterations in saliva may be a result of medications (at least 400 drugs may induce xerostomia. Diuretics, antihypertensives, antipsychotics, antihistamines, antidepressants, anticholinergics, antineoplastics, amphetamines, barbiturates, hallucinogens, cannabis, and alcohol have been associated with a reduction in salivary flow and may lead to oral problems like progressive dental caries, fungal infection, oral pain, and dysphagia [92,93].

Qualitative changes in salivary composition can also provide diagnostic information concerning oral problems: increased levels of albumin in whole saliva were detected in patients who received chemotherapy as treatment for cancer and subsequently developed stomatitis, reduced salivary EGF levels may be important for the progression of radiation–induced mucositis, higher levels of salivary nitrate and nitrite, and increased activity of nitrate reductase, were found in oral cancer patients compared with healthy individuals, and were associated with an increased odds ratio for the risk of oral cancer [94–96]. 

Saliva is also very suitable for the monitoring of oral bacteria that can survive in saliva, and can utilize salivary constituents as a growth medium, for example, increased numbers of *Streptococcus mutans* and Lactobacilli in saliva were associated with increased caries prevalence and with the presence of root caries, detection of certain bacterial species in saliva can reflect their presence in dental plaque and periodontal pockets [97– 103]. The changes of the components of the saliva may also be used for periodontal diagnosis [ 104–106]. Recent studies focus on the potential role of periodontal disease as a risk factor for cardiovascular and cerebrovascular diseases as a possible link with metabolic syndrome and oxidative stress [107–111].

**Uric acid** levels are significantly lower in saliva from heavy smokers, oral cancer, periodontitis and diabetic patients with periodontitis, giving emphasis of oxidative stress in oral cavity. A lower level of saliva **albumin** was found in periodontal disease and diabetic patients, probably due to oxidative stress in the oral cavity.

**Total antioxidant capacity (TAC)** is significantly lower in periodontal disease, diabetics, smokers, and oral cancer. When exposed to cigarette smoke, saliva loses its TAC and becomes a potent prooxidant milieu.

Salivary peroxidase is lower in smokers, most probably due to the cyanide ions present in cigarette smoke, a very powerful inhibitor of hem peroxidase and in saliva from patients with oral cancer. The results indicate that exposure to cigarette smoke (CS) caused a statistically significant decrease of salivary **gamma–glutamyltransferase (GGT)** caused by the oxidative stress. **Salivary aspartate aminotransferase (AST)**, **alanine aminotransferase (ALT)** and **alkaline phosphatase (ALP)** are markers in periodontitis [21–111].

In **Alzheimer disease (AD)**–Salivary AChE activity may prove to be a useful marker of AD–associated changes in central cholinergic activity and the responsiveness of patients to treatment with AChE inhibitors [1].

## 

**Bone Turnover Markers in Saliva?** Saliva may be a valuable tool for assessing human markers of bone turnover and it has a great impact on dental implants and osteoporosis. Further research is necessary to determine whether salivary levels of bone turnover markers correlate with serum and/or urine measures [112].

**Current National Initiatives for Salivary Diagnostics**. From 2001 to 2004, the National Institute for Dental and Craniofacial Research invested 52 million dollars in salivary diagnostic research to concurrently spearhead the development of technologies to virtually detect any analyte in saliva as well as to comprehensively identify all the proteins in saliva.

**Saliva Testing in the Genomic Era of Medicine**–As we enter the era of genomic medicine therefore salivary diagnostics will play an increasingly important role in the early detection of disease, the monitoring of disease progression, and the evaluation of patient behavior including treatment compliance and lifestyle choice. However, about the **sialochemistry** in the twentieth century the best conclusion is perhaps in the title of a review written by Irwin Mandel, ‘Salivary Diagnosis: Promises,Promises’ or the title of a guest editorial by Daniel Malamud in the Journal of the American Dental Association in 2006: ‘Salivary diagnostics. The future is now’

We are likely to see the increased utilization of saliva as a diagnostic fluid and in consequence, dentists will have greater involvement in the identification and monitoring of certain non–oral disorders. 
